# Spatial distribution and the imbalance between supply and demand: an analysis of the geographical characteristics and regional differences of elderly care institutions in China

**DOI:** 10.1186/s12942-025-00445-3

**Published:** 2025-12-31

**Authors:** Kexin Zhang, Tingzhi Miao, Tiangui Wang, Huiqing Han, Jiaoting Peng, Yan Ji

**Affiliations:** 1 School of Management Science and Engineering, Guizhou University of Finance and Economics, Guiyang, 550025 Guizhou China; 2 College of Architecture and Urban Planning, Guizhou Institute of Technology, Guiyang, 550003 Guizhou China; 3 Research Office, Guizhou University of Finance and Economics, Guiyang, 550025 Guizhou China

**Keywords:** Elderly care institutions, Spatial distribution, Influencing factors, Optimal-parameters-based geographical detector, China

## Abstract

Against the backdrop of China’s continuously intensifying population aging, the spatially balanced distribution of elderly care institutions (ECIs) has emerged as a critical issue for alleviating elderly care pressure and advancing social equity. Utilizing nationally registered ECI data, this study integrates ArcGIS spatial analysis with an Optimal-Parameter Geographical Detector (OPGD) approach to systematically investigate the spatial heterogeneity, supply-demand imbalance patterns, and underlying formation mechanisms of ECIs in China at the provincial level. A key finding is the pronounced spatial and structural imbalance between supply and demand. Kernel density estimation reveals a multi-level clustering structure centered on Shanghai and Chongqing, while the consistency coefficient identifies distinct mismatch patterns: regions such as Xinjiang and Northeast China experience “supply exceeding demand,” whereas economically dynamic areas like the Pearl River Delta face “supply falling behind demand.” Spatially, ECIs overall follow a “dense southeast–sparse northwest” pattern closely aligned with the “Hu Huanyong Line,” with six provinces including Henan and Sichuan accounting for 34.1% of institutions, compared to only 1.6% in four western provinces/regions and Hainan. Furthermore, OPGD analysis identifies the permanent population size and number of hospital beds as the dominant factors influencing the spatial layout of ECIs. Their interaction with public transportation accessibility and fiscal expenditure significantly enhances explanatory power, highlighting the crucial role of medical-care integration and government investment in resource allocation. This study provides a scientific basis for optimizing the spatial allocation of elderly care resources and promoting coordinated regional development in China.

## Introduction

According to the 2023 National Report on the Development of Aging, China’s population aged 60 and above reached 297 million (21.1% of the total), while those aged 65 and above reached 217 million (15.4%), marking the country’s full entry into an aging society [1]. The China Statistical Yearbook 2024 and local government statistics data show that 19 of China’s 31 provincial-level regions are experiencing moderate or severe aging, with Liaoning (21.1%) and Shanghai (19.6%) having the highest proportions of residents aged 65 and above. In response, the Third Plenary Session of the 20th Central Committee of the Communist Party of China has proposed active measures to address population aging. Key initiatives include refining elderly care policies and mechanisms, optimizing the provision, improving the management of public elderly care institutions (ECIs), and encouraging greater participation from enterprises and social actors. These efforts are aimed at establishing a diversified and multi-tiered elderly care system to mitigate the social pressures associated with a growing aging population [2, 3]. The “Guidelines on Deepening the Reform and Development of Elderly Care Services,” a landmark document in China’s elderly care policy, were jointly issued by the Central Committee of the Communist Party of China (CPC) and the State Council on December 30, 2024, representing the first specialized policy released under their joint name. The Guidelines emphasize accelerating the building of a comprehensive elderly care service network, optimizing supply structure based on “home-based care as the foundation, community care as the support, and institutional care as the backbone”, and strengthening the urban-rural three-tier service system integrating home, community, and institutional care [4]. As a top-level design for a modern elderly care service system, it addresses the urgent needs of the national strategy to actively respond to population aging and provides fundamental policy guidance for China’s elderly care service reform. Yet, amid social progress and shrinking family sizes, traditional home-based care can no longer meet growing demands; diversified, innovative, high-quality elderly care models have become an inevitable future trend [5]. While community-based care is promoted nationwide, it still faces challenges: scarce professional nursing resources, low service standardization, and a fragmented system unable to meet diverse elderly needs. As a core part of the elderly care service system, institutional elderly care offers comprehensive daily care and long-term medical support, gradually becoming indispensable to China’s social elderly care system. Under the national strategy of actively responding to population aging, the 2025 Government Work Report identified the construction of the elderly care service system as a key annual task and proposed to “improve development policies and mechanisms for elderly care services and industries, support the establishment of professional ECIs, and encourage social forces to develop large-scale, specialized facilities integrating medical and care services.” Enhancing standardization and resource integration in institutional elderly care, along with establishing a multi-tiered service system, is essential to mitigate structural supply-demand imbalances and effectively respond to population aging.

Current research on ECIs and facilities by domestic and international scholars primarily focuses on three core areas: facility spatial characteristics [6–13], institutional accessibility [14–16], and supply-demand measurement [5, 17, 18]. Dominant methods include kernel density estimation, nearest neighbor index, spatial cluster analysis, and the two-step floating catchment area method [19]. To enhance equity in cross-regional elderly care provision, existing quantitative research on ECIs primarily focuses on two spatial scales: prefecture-level cities (emphasizing small-region analysis) and provincial regions [14–22]. At the provincial scale, constrained by China’s ECI development patterns, research tends to concentrate on specific institution types like nursing homes, utilizing tools such as the Gini coefficient to analyze regional distribution patterns [20, 21]. For the prefecture-level city scale, studies center on spatial planning and accessibility [19], investigating how intra-urban population distribution, economic zoning, transportation infrastructure, and user preferences shape ECI layouts [22]. As a core component of the elderly care service system, the spatial distribution and service capacity of ECIs not only directly impact the quality of life of older adults but also reflect the equity of public resource allocation. However, existing municipal-level studies, while valuable for local insights, lack comparable metrics across cities of varying sizes and development levels, hindering a systematic understanding of ECI distribution patterns at the national scale. To systematically reveal the agglomeration or diffusion effects resulting from spatial scale differences—such as the root causes of interregional supply-demand imbalances and the patterns of cross-provincial resource allocation—a national-scale comparative research perspective is essential. This perspective is key to addressing current research gaps and crucial for coordinating the allocation of elderly care resources nationwide and promoting equitable distribution across regions.

However, limited by data availability and completeness, no study has yet systematically examined the spatial distribution characteristics of China’s ECIs at the national level. Research remains particularly underdeveloped regarding geospatial distribution patterns, supply-demand imbalances, and their underlying drivers. To address this gap, this study adopts a geographical perspective, employing web scraping technology to compile national ECI data and applying ArcGIS spatial analysis to examine geospatial disparities of these institutions across China. Furthermore, we utilize the optimal-parameter geographical detector to identify the factors influencing these spatial disparities. This national-scale analytical framework will provide government agencies with macro-level insights into regional variations and support evidence-based planning that aligns institutional distribution with local aging trends. Ultimately, this research aims to inform strategic resource allocation and promote high-quality development in China’s elderly care service industry.

## Data and methods

### Data

The data on registered ECIs selected for this study were are sourced from ECIs registered with the Ministry of Civil Affairs of the People’s Republic of China (https://zwfw.mca.gov.cn/), with a cut-off date of December 31, 2024. These ECIs primarily include elderly welfare homes, elderly care homes, nursing homes, social welfare institutions (for the elderly), senior apartments, and other types. A total of 41,825 ECIs across 31 provincial-level regions (excluding Hong Kong, Macao, and Taiwan) in China were selected as research subjects. The names or addresses of these institutions were entered into the Baidu Map Picker Coordinate System (https://api.map.baidu.com/) to obtain their geographic coordinates. These coordinates were then converted into vector data for the institutions using the ArcGIS 10.8 platform. Socioeconomic data—including the resident population (10,000 persons), proportion of population aged 65 and above (%), old-age dependency ratio (%), number of employed persons (10,000 persons), general public budget expenditure (100 million yuan), GDP (100 million yuan), GDP per capita (yuan), number of operational buses and trolleybuses (units), disposable income per capita (yuan), number of beds in medical institutions (units), and urbanization rate for each province—were obtained from the China Statistical Yearbook 2024, China Urban-Rural Construction Statistical Yearbook 2023, and 2024 editions of provincial statistical yearbooks. All datasets refer to the year 2023, ensuring temporal consistency. The analysis covers 31 provincial-level units in China, excluding Hong Kong, Macao, and Taiwan.

### Methods

This study utilized the Baidu API geocoding service to obtain the longitude and latitude coordinates of ECIs nationwide. Multiple analytical methods were employed to systematically investigate the spatial patterns and formation mechanisms of ECIs in China. The methodological design follows a clear macro-meso-micro hierarchical logic, with each method performing complementary roles to ensure comprehensive coverage of the research scope. At the macro level, the Geographic Concentration Index [23, 24] and Imbalance Index [25] were applied to assess the overall provincial-scale distributional equity and structural concentration of ECIs. The Consistency Coefficient [23, 24] was further introduced to evaluate the supply–demand matching relationship between ECI distribution and the aging population, addressing a key dimension of spatial mismatch. At the meso scale, Kernel Density Estimation (KDE) and Hot and Cold Spot Analysis [25] were employed to identify regional agglomeration patterns and significant spatial clusters of ECIs, revealing core density zones and spatially correlated high- and low-value areas. At the micro-mechanism level, the study applied the Optimal-Parameter Geographical Detector (OPGD) [26, 27] to quantify the driving factors behind the observed spatial heterogeneity. Given that OPGD requires discrete independent variables, this study first processed continuous variables (e.g., elderly population density, fiscal expenditure) through systematic discretization: the OPGD method tests multiple discretization approaches for each variable to determine the optimal scheme (including interval count and discretization method), thereby improving the accuracy of factor detection [3]. Using the GD package in R language, we identified optimal discretization parameters via grid search, then applied the factor detector and interaction detector modules to clarify key influencing factors (e.g., permanent population size) and their synergistic effects. Detailed analytical methods are summarized in Table 1.

**Table 1:** Description of research methods and their geographical significance

Serial number	Methods	Formula	Parameter description	Geographical significance
(1)	Kernel Density Estimation		(*x* - *xi*) denotes the Euclidean distance between the estimation point *x* and the observed event location *xi*, where *h* > 0 is the bandwidth parameter	Kernel Density Estimation (KDE) is a technique for characterizing the spatial variation in density of point features within a defined neighborhood [25]
(2)	Geographic concentration		*P*_*i*_ and *R*_*i*_ represent the geographic concentration of the elderly population aged 65 and above and elderly care institutions in province *i*, respectively; *p*_*i*_ and *r*_*i*_ denote the number of elderly individuals aged 65 and above and the number of elderly care institutions in region *i*, while *t*_*i*_ represents the area of region *i*	Geographic concentration is a metric that synthesizes the agglomeration level of elements within a region, utilized here to analyze the relationship among the elderly population, elderly care institutions, and the regional area [23, 24]
(3)	Consistency coefficient		The parameters have the same meaning as in the Geographic Concentration formula (1). *CC* reflects the degree of consistency between elderly care institutions and the population aged 65 and above in a given region [23, 24]	*CC* > 1 indicates that the agglomeration level of elderly care institutions in that region is higher than the concentration of the elderly population distribution. Conversely, *CC* < 1 means that the agglomeration level of elderly care institutions is lower than the concentration of the elderly population distribution
(4)	Disparity index		*n* is the number of regions; *Yi* is the cumulative percentage of the *i*_*− th*_ rank in descending order of the proportion of elderly care institutions in each province and municipality relative to the national total	*S* is the disparity index. If *S* = 0, it indicates a perfectly even distribution; if *S* = 1, it signifies a highly uneven distribution [25]
(5)	Hot Spot Analysis (Getis-Ord Gi*)		*xⱼ* is the attribute value of spatial feature *j*; *w* is the spatial weight between features *i* and *j*, defined as: 1 if they are adjacent, and 0 if they are not; *n* is the total number of spatial features; *X̄* is the mean value of the spatial features; *S* is the standard deviation of the spatial features; The Gi* statistic is expressed as a z-score	By comparing the local sum within a feature’s neighborhood to the global sum, it quantifies the level of local spatial association between that feature and its neighbors. This facilitates the detection of statistically significant clusters of high values (hot spots) and low values (cold spots) [24, 25]
(6)	Optimal parameter geographical detector		*h* denotes the stratum of variable *Y* or factor *X*; *N*_*h*_ and *N* are the number of units in stratum and the entire region, respectively; *σ²* and *σ*_*h*_^2^ are the variances of the *Y* values for the entire population and for stratum *h*, respectively	The *q* statistic is a measure used to quantify spatial heterogeneity and to identify the interactive effects between explanatory factors and the dependent variable. A higher *q* value signifies that the corresponding factor exerts a greater influence on the spatial distribution pattern of elderly care institutions, whereas a lower value indicates a weaker influence [26, 27]

All research methods in this study are conducted using provincial-level geographical units

## Results

### Interprovincial spatial differentiation characteristics of elderly care institutions in China

**Fig. 1:**
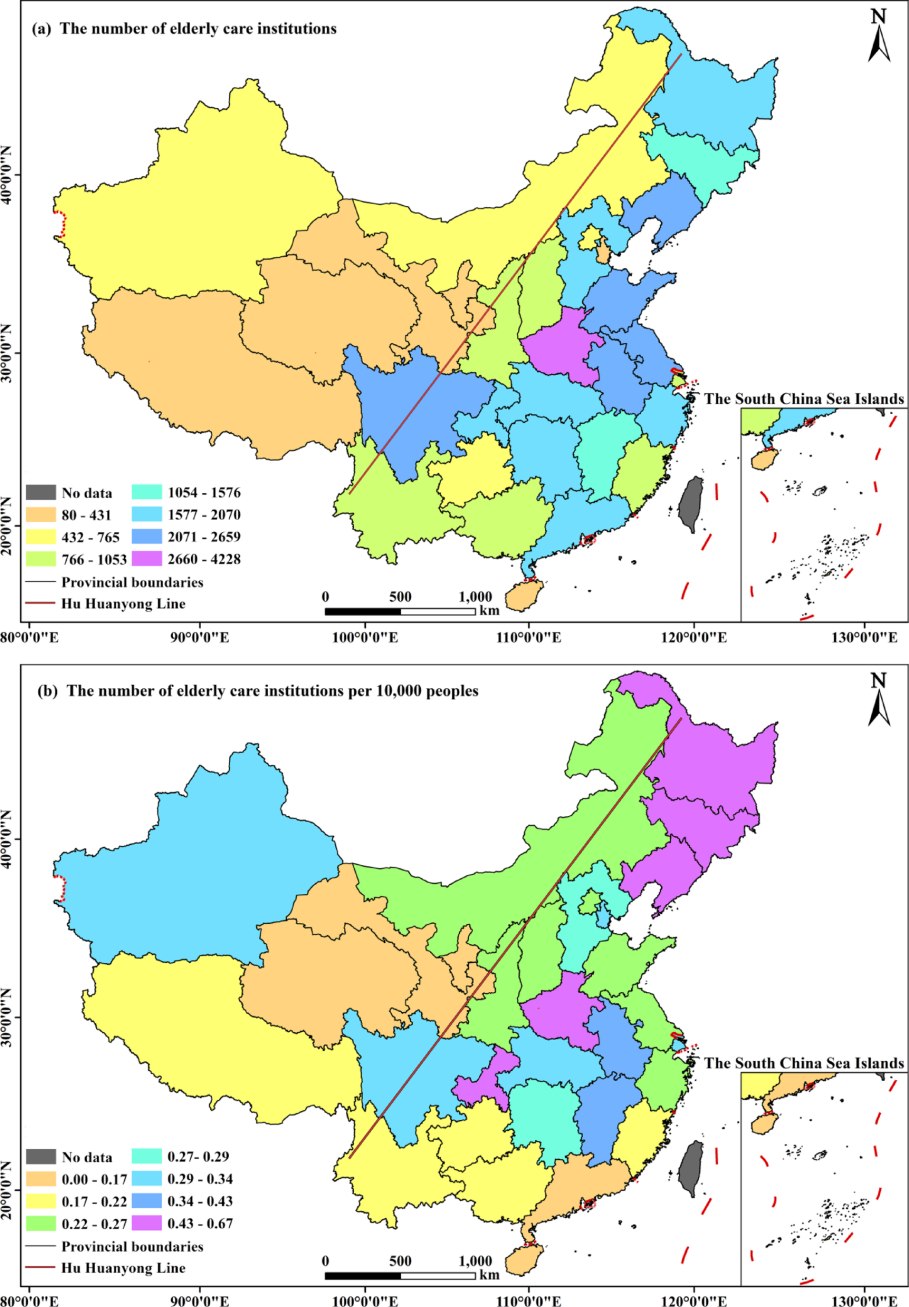
Spatial distribution of the number of elderly care institutions (**a**) and per 10,000 persons (**b**) in various provinces of China

The spatial distribution of ECIs in China reveals a pronounced east–west disparity, closely following the Hu Huanyong Line, with the majority concentrated in the southeastern region (Fig. 1). In terms of absolute numbers, six provinces—Henan (4,228), Sichuan (2,659), Anhui (2,592), Shandong (2,472), Liaoning (2,315), and Jiangsu (2,251)—collectively account for 34.1% of all ECIs nationwide. In contrast, five western provinces and regions, including Xizang (80), Ningxia (97), Qinghai (98), Hainan (138), and Gansu (342), together contribute only 1.6% of the total. When examined on a per capita basis (ECIs per 10,000 people), the pattern becomes more nuanced (Fig. 1b). The three northeastern provinces (Liaoning, Jilin, Heilongjiang) and Chongqing show the highest per‑capita availability (exceeding 0.54), largely due to accelerated population aging driven by youth out-migration and a historically developed institutional base. By contrast, western provinces such as Gansu and Qinghai exhibit low per‑capita supply (below 0.14), constrained not only by limited fiscal resources but also by the high unit cost of serving dispersed populations. Notably, despite its economic strength, Guangdong also shows low per‑capita availability, reflecting pressures from land scarcity and a large influx of younger migrants, which dilute resource allocation for the local elderly. In summary, supplementing absolute ECI counts with per‑capita metrics shifts the analytical focus from sheer quantity to relative adequacy. The results illustrate that spatial disparities are not merely a function of population density, but arise from the complex interplay of demographic change, economic capacity, and migration dynamics.

**Fig. 2:**
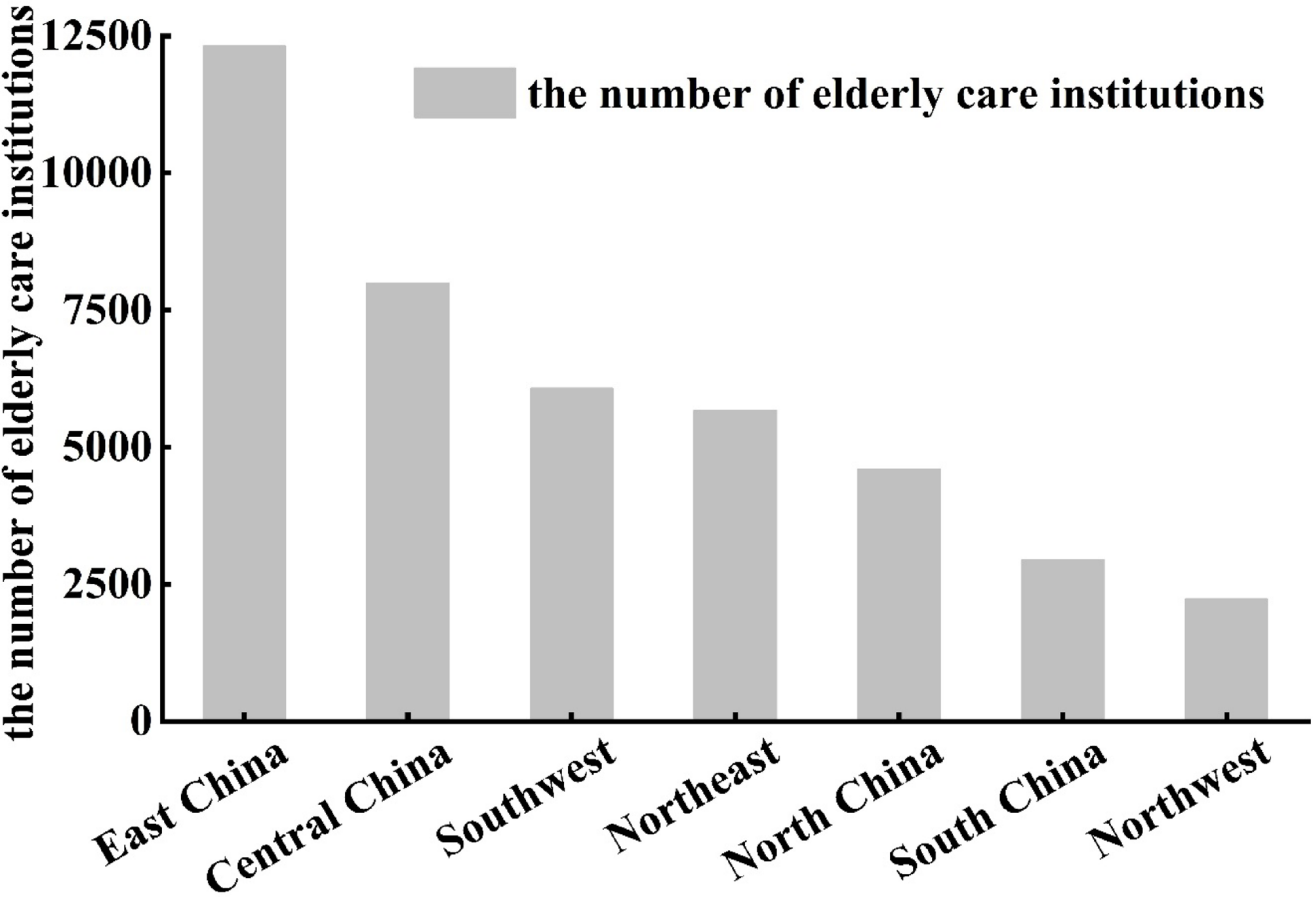
Distribution characteristics of the number of elderly care institutions according to seven geographical divisions in China

Analyzing from the perspective of China’s geographical divisions (Fig. 2), the distribution is highly uneven. East China hosts the largest share of ECIs (12,315 institutions, 29.4% of the national total), followed by Central China (19.1%), Southwest (14.5%), and Northeast (13.6%). In contrast, Northwest and South China account for only 5.3% and 7.1%, respectively. This macro-level pattern is strongly influenced by the underlying east-dense, west-sparse population distribution and the more robust economic foundations in eastern regions, which support a more comprehensive elderly care service system. Building on this regional framework, the provincial-level analysis reveals more granular patterns and exceptions (Fig. 1a). For instance, within the high-density East China region, Jiangsu and Shandong are major contributors, whereas within the less dense Northeast region, Liaoning stands out with a notably high number of ECIs. This finer-scale variation underscores that while regional trends are informative, significant disparities exist at the sub-regional and provincial levels. The analysis of ECIs per 10,000 people further refines this understanding, identifying provinces like Guangdong—located within the high-supply East China region—as having unexpectedly low per capita availability, highlighting unique local pressures such as a large migrant youth population.

### Spatial agglomeration patterns of elderly care institutions in China

**Fig. 3:**
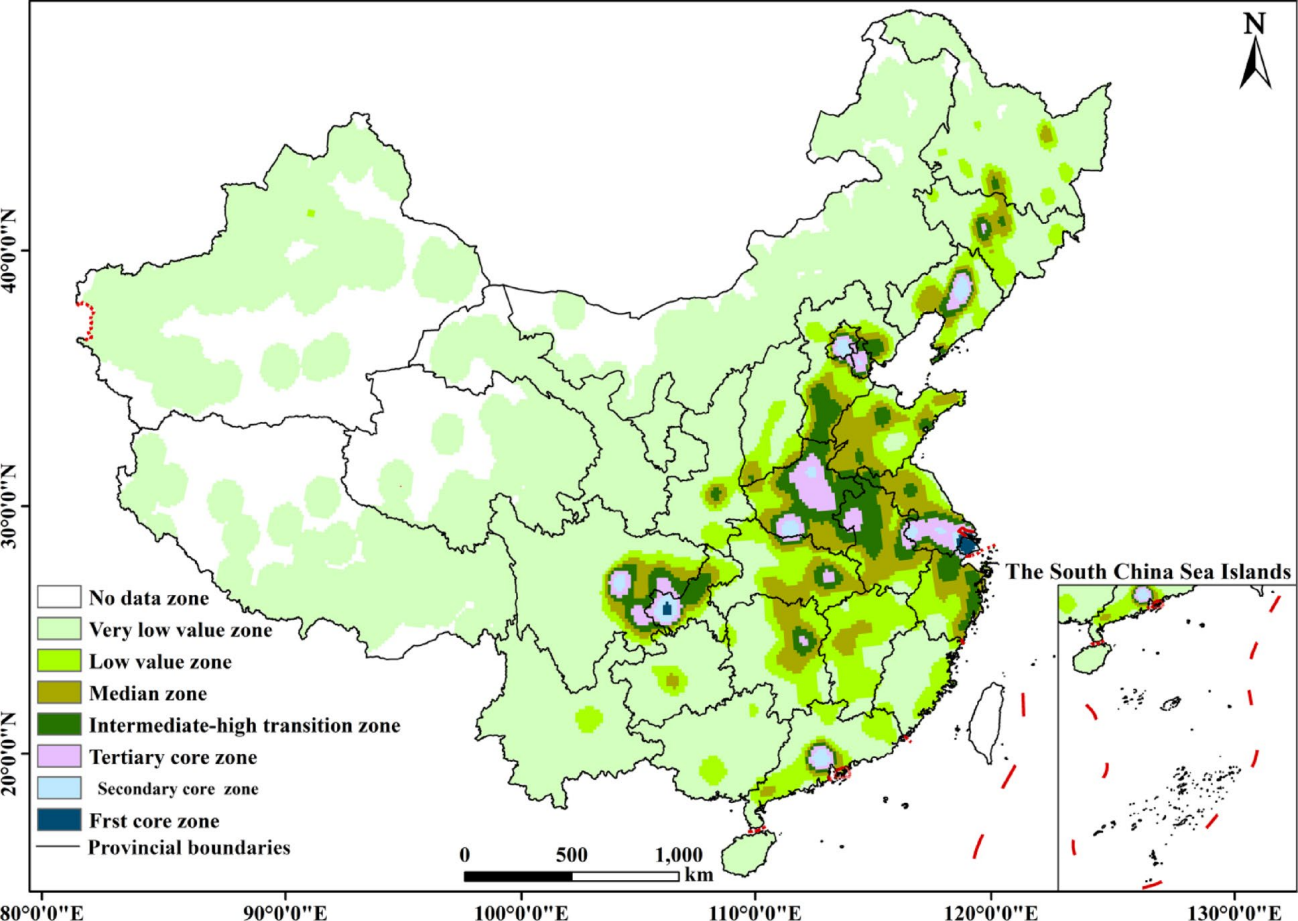
Kernel density of elderly care institutions in China

Employing the Kernel Density Estimation (KDE) method to investigate the spatial distribution types of ECIs in China, the density values were classified using the Natural Breaks Classification (Jenks) method into eight categories: First core zone, Secondary core zone, Tertiary core zone, Low-value zone, Relatively Low- value zone, Medium- value zone, Medium-to-high value transition zone, and No-value zone (Fig. 3). The spatial distribution of ECIs in China exhibits multiple agglomeration cores, including 2 First core zones, 8 Secondary core zones, and 8 Tertiary core zones, all located in the southeastern part of the country. The First core zones are primarily situated in Shanghai and Chongqing. The Secondary core zones are mainly distributed across provinces and municipalities such as Sichuan, Chongqing, Guangdong, Shanghai, Liaoning, Henan, Beijing, and Tianjin. The distribution range of the Tertiary core zones is similar to that of the Secondary core zones, extending outward from them and radiating to drive development in surrounding areas. The First, Secondary, and Tertiary core zones collectively form an approximately “*T* " - shaped distribution pattern. Simultaneously, these core zones connect with medium and low-value areas, creating a patchy spatial gradient that decreases radially from the Primary core zones outwards towards the medium and low-value areas. The Low-value and Medium-value zones are predominantly distributed in Northwest and Southwest China, appearing in block-like and areal patterns. This pattern stems from several factors: The First core zones (Shanghai, Chongqing) leverage their status as direct-controlled municipalities, benefiting from economic agglomeration effects and policy advantages, resulting in a well-established foundation for ECIs. The Secondary core zones often consist of populous provinces (e.g., Henan), economically strong provinces (e.g., Guangdong), and old industrial bases (e.g., Liaoning), characterized by high population density, pressing aging-related demands, and strong fiscal support capacity. The “*T* " - shaped pattern highly resembles the alignment of the eastern coastal economic belt and the Yangtze River economic belt. Conversely, the Low-value zones in the Northwest and Southwest are constrained by less developed economies, sparse populations, geographical barriers, and relatively limited policy support and resource allocation. These factors have led to a lag in the development of ECIs, resulting in a peripheral declining trend.

### Spatial equilibrium in the distribution of elderly care institutions across China

**Fig. 4:**
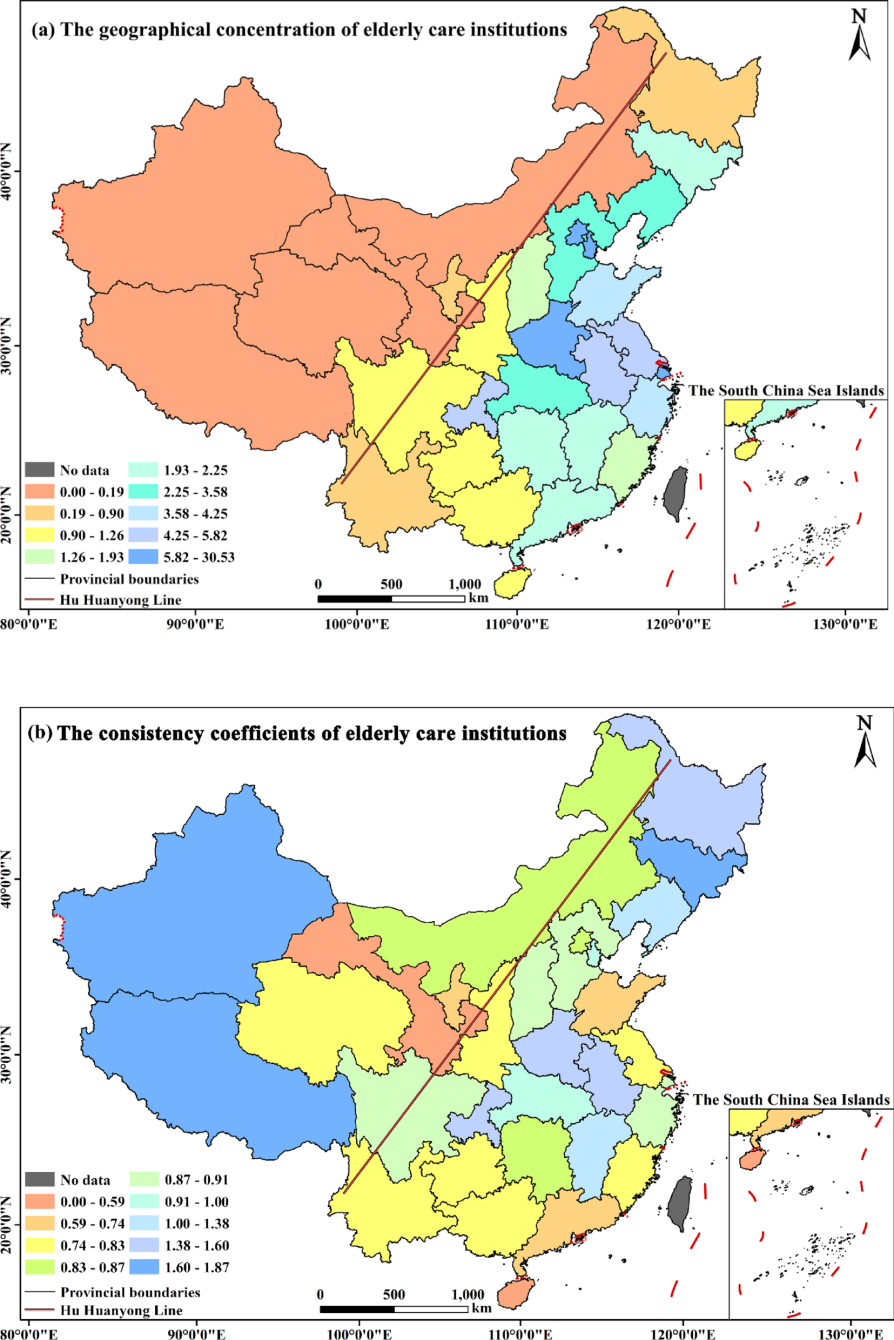
Spatial distribution of geographical concentration of elderly care institutions and their consistency coefficients in provincial-level regions of China

Based on the geographic concentration index, consistency coefficient (CC), and imbalance index (S), this study reveals a highly concentrated and uneven spatial distribution of ECIs across China. Facilities are more abundant in socioeconomically developed eastern regions, while western and southwestern areas with lower economic development show relative scarcity, leading to substantial regional disparities (Fig. 4a). The distribution exhibits a pronounced disparity, closely following the “denser southeast–sparser northwest” pattern of the Hu Huanyong Line. At the provincial level, Shanghai, Beijing, and Tianjin exhibit significantly higher concentrations of ECIs, whereas northwestern (e.g., Qinghai, Gansu, Inner Mongolia, Ningxia, Xinjiang) and southwestern (Yunnan, Xizang) regions all fall below 0.5. For instance, Shanghai and Beijing—the two highest-concentration regions—accommodate approximately 170 and 40 times more facilities per unit measure than Xinjiang, Gansu, and Ningxia, respectively. The consistency coefficient (*CC*) further assesses alignment between the spatial distribution of ECIs and the elderly population. Regions with *CC* > 1—including Xinjiang, Xizang, Henan, Jiangxi, and the three northeastern provinces—suggest “supply ahead of demand” (Fig. 4b). This can be attributed to policy support such as national strategies for border revitalization and fiscal transfers in western regions, as well as aging-adapted subsidies and resource reallocation in the northeast [28]. By contrast, eastern coastal and central provinces with *CC* < 1 demonstrate a clear misalignment between the supply of care institutions and the pace of population aging. In highly urbanized and economically advanced zones like the Yangtze River Delta and Pearl River Delta, the rapid concentration of elderly residents has not been met with parallel growth in care facilities, largely due to constraints such as scarce land resources and high operating costs. Less developed provinces including Yunnan and Guizhou, hampered by limited fiscal investment, also fall below the national average in terms of care facility availability. Additionally, the imbalance index (S), calculated via Formula (4), yields a value of 0.39 (0 < *S* < 1), indicating a moderate structural imbalance in the interregional distribution of elderly care resources—further supporting the observed spatial inequality.

**Fig. 5:**
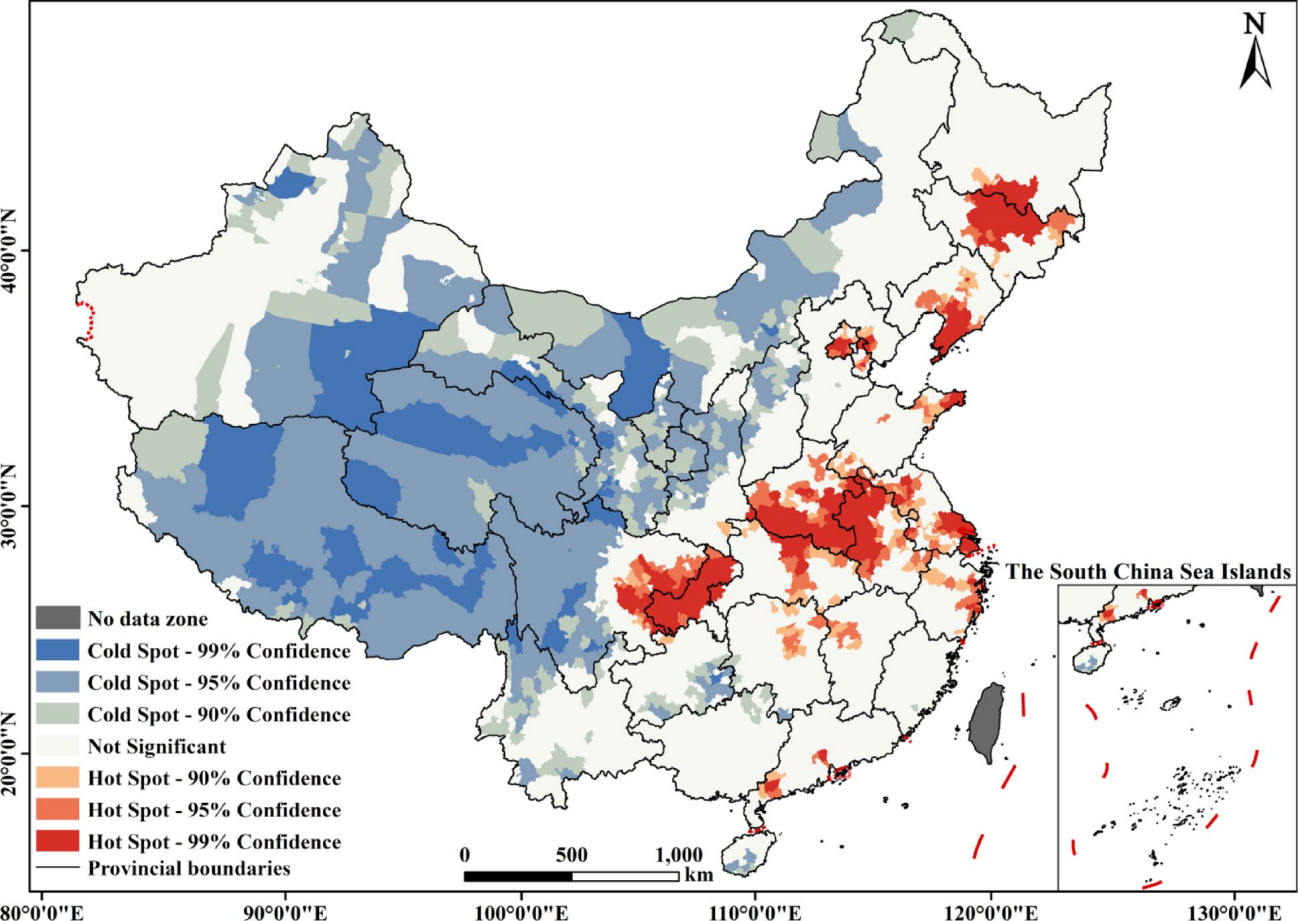
Hot-cold spot distribution of elderly care institutions in China

Finally, the Getis-Ord Gi* analysis in ArcGIS was employed to identify hot and cold spots of ECI agglomeration in China (Fig. 5). Overall, their spatial pattern shows regional linkage and marked imbalance, reflecting the tight coupling between the “core-periphery” hierarchy and geographical, socioeconomic, and policy factors. Hot spots concentrate in eastern/central China (Shanghai, Anhui, Henan), southwest China (Chongqing), and northeast China (Liaoning, Jilin) (Fig. 5). Sub-hot spots (e.g., Hubei) and transition zones surround main hot spots, forming a roughly " *T* “-shaped distribution. Pension policy spillover from urban agglomerations like Chengdu-Chongqing and Beijing-Tianjin-Hebei to neighboring provinces (Shaanxi) drives sub-hotspot formation. These areas typically boast advanced economies (Shanghai), large populations (Henan, Anhui), or high aging rates (Liaoning, Jilin, Chongqing), where strong economic foundations and elderly care demand jointly fuel ECI agglomeration.

In contrast, cold spots are predominantly located in the five northwestern provinces, Xizang, and parts of Yunnan and Sichuan. These regions face multiple constraints, including economic underdevelopment, limited fiscal capacity, and low density of elderly residents, which, combined with high operational costs, lead to sparse facility coverage.

This spatial disparity reflects a “Matthew Effect” in elderly care resource allocation: developed regions attract more resources through market and policy advantages, while underdeveloped areas remain trapped in a “low-level equilibrium”. To break this cycle, a “precision identification-dynamic compensation-regional coordination” mechanism is needed—leveraging big data for cold spot demand monitoring, targeted fiscal transfers for capacity building, and urban agglomeration resource sharing to gradually soften rigid spatial divides.

## Influencing factors of spatial differentiation of elderly care institutions in China

### Selection of influencing factor indicators and theoretical analysis

**Fig. 6:**
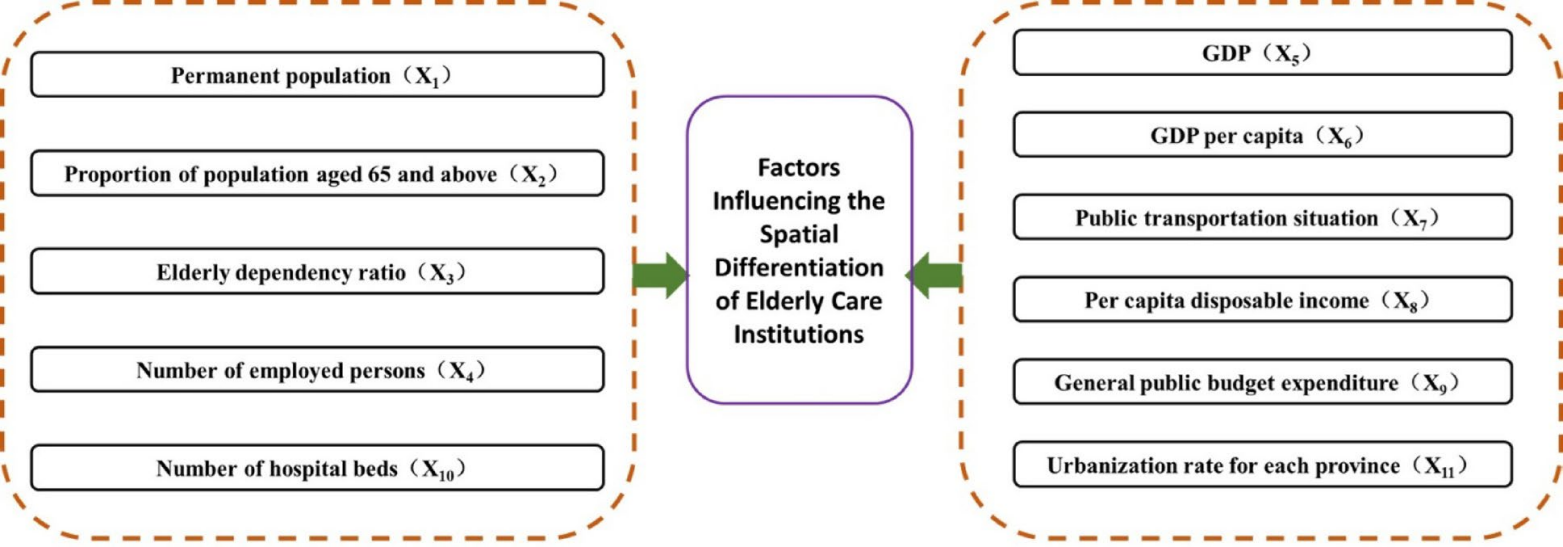
Analysis framework of influencing factors for the elderly care institutions

To investigate the factors influencing the spatial distribution of ECIs in China, this study selects 11 socioeconomic variables (Fig. 6). The selection was mainly based on the core conclusions of existing relevant studies on the spatial distribution of elderly care institutions (e.g., nursing homes, elderly care homes, and welfare homes) [3, 5, 8, 18, 31], combined with the regional actual characteristics of elderly care institution development in China (such as administrative management systems, differences in population aging, etc.). Specifically, based on the functional logic of the 11 variables, they are grouped into 8 categories of influencing mechanisms. (1) The elderly population constitutes the primary target group of ECIs. A higher proportion of older adults and a greater old-age dependency ratio are likely to increase the demand for elderly care services, leading to a denser spatial distribution of such facilities. (2) GDP per capita and disposable income per capita reflect the average living standards. Higher values indicate better provision of public services—including infrastructure, education, and healthcare—which may support the development of ECIs. (3) With rising labor force participation and a growing employed population in China, the tension between work responsibilities and elder care has become more pronounced [29]. Consequently, regions with higher employment rates may exhibit greater demand for institutional elderly care services. (4) Regions with higher general public budget expenditure are often able to allocate more financial resources toward the construction and operation of elderly care facilities, resulting in a relatively larger supply of such institutions. (5) The number of medical institution beds serves as an indicator of regional healthcare capacity. While situating ECIs near dense medical resources offers convenience for older adults with healthcare needs, it also intensifies concerns regarding equitable spatial distribution and accessibility for residents in underserved areas. (6) Public transportation accessibility, reflected in the number of operational buses and trolleybuses, influences the ease of reaching elderly care facilities. Better transport infrastructure can enhance the accessibility and attractiveness of these institutions. (7) Although not explicitly listed among the 11 variables in this excerpt, the urbanization rate—often included in such analyses—represents the level of urban development. More urbanized regions typically possess more advanced infrastructure, including elderly care facilities. (8) Higher levels of regional economic development are associated with increased demand for high-quality elderly care services and greater public acceptance of formal care institutions. As a result, economically developed areas, often indicated by higher GDP, may host a larger number of ECIs.

### Factor detection analysis of the influencing factors for elderly care institutions

Using the number of ECIs as the dependent variable and 11 influencing factors as independent variables, this study applied different discretization methods and obtained optimal spatial discretization results for the independent variables through the OPGD method (Table 2). Based on the *q*-values derived from factor detection, the following factors influencing the spatial distribution disparities of ECIs in China were identified, ranked in descending order of explanatory power: number of hospital beds > resident population > public transportation accessibility > size of the employed population > government fiscal expenditure > GDP > old-age dependency ratio > proportion of population aged 65 and above > urbanization rate > GDP per capita > disposable income per capita. Among these 11 independent variables, six—resident population, proportion of population aged 65 and above, number of employed persons, number of operational buses and trolleybuses, number of beds in medical institutions, and general public budget expenditure—have explanatory power exceeding 30% and passed the significance test at the 1% level (Table 2), indicating that they are important factors influencing the spatial differentiation of ECIs. Although GDP and the old-age dependency ratio exhibit relatively high *q*-values (0.5799 and 0.4336, respectively), their *p*-values > 0.01 suggest that while they influence the spatial differentiation of ECIs, the effect is not statistically significant. The remaining three factors—GDP per capita, urbanization rate, and disposable income per capita—have the least impact on the spatial differentiation of ECIs, each with explanatory power below 30%. The number of medical institution beds, representing the density of regional medical resources, exerts the strongest influence on ECIs. Currently, China has approximately 35 million disabled and semi-disabled elderly individuals [30] who require accessible medical support. The service quality and market competitiveness of ECIs are directly constrained by the availability of medical resources, making such resources a “rigid constraint” on the spatial distribution of these institutions.

As a core indicator for assessing elderly care demand, the size of the resident population plays a crucial role in influencing ECIs. In densely populated regions such as the Yangtze River Delta and Pearl River Delta, aging-related pressures continue to intensify, and the trend toward smaller family structures further drives a significant increase in demand for institutional care. Public transportation accessibility is a key consideration for the elderly when selecting care facilities, influencing occupancy rates and, in turn, the operational sustainability of ECIs. Cities with high densities of subway and bus stations can reduce visitation costs for family members, extend the service radius of ECIs, and improve occupancy levels. The scale of the employed population indirectly reflects regional economic vitality and household payment capacity. Residents in regions with higher employment rates generally enjoy relatively higher income levels, enabling them to afford market-based elderly care services and thereby fostering the development of ECIs. General public budget expenditure reflects the government’s capacity to invest in public services. Regions with higher expenditures typically have more resources for the construction of elderly care service facilities and subsidies, which is more conducive to the establishment and layout of ECIs.

As a key indicator of regional economic strength, GDP influences the extent of social capital involvement and thus affects the geographical distribution of ECIs. However, in more economically developed eastern regions with mature market mechanisms, fully market-oriented ECIs account for a larger share. This is partly because market capital tends to flow into high-end elderly care rather than universal care institutions, leading to a “structural imbalance” in economically strong provinces—where high-end care is oversupplied, while universal care remains insufficient. Although regions with high urbanization rates exhibit concentrated demand and abundant infrastructure and public service resources, elevated land costs often drive ECIs to suburban areas, reducing transportation convenience and creating a spatial mismatch between supply and demand. The study by Li et al. [31] also confirmed a negative correlation between urbanization rate and the spatial distribution of ECIs. GDP per capita and disposable income per capita have the least impact on the spatial distribution of ECIs, primarily because China’s ECI sector still relies heavily on financial subsidies and universal care provision. Moreover, in policy and planning related to ECI construction, the government tends to prioritize factors that directly affect institutional accessibility and service quality—such as population distribution, number of hospital beds, and public transportation accessibility. According to some local regulations, the monthly fee for universal ECIs must not exceed 1.3 to 1.5 times the previous year’s average monthly disposable income per capita. At present, the average monthly fee for universal ECIs in Shenzhen is approximately 7,000 yuan [32], which constrains the role of individual payment capacity. Therefore, the influence of individual economic indicators on ECIs is moderated by policy interventions.

**Table 2:** Classification and factor detection results of influencing factors

Variables	Discretization methods	Number of intervals	q-value	*p*-value
X_1_	Natural Breaks (Jenks)	5	0.6968	0.0004
X_2_	Geometric Interval	4	0.3238	0.0079
X_3_	Quantile	4	0.4336	0.0103
X_4_	Geometric Interval	5	0.6547	0.0001
X_5_	Equal Interval	5	0.5799	0.0339
X_6_	Quantile	5	0.2041	0.2811
X_7_	Natural Breaks (Jenks)	5	0.6651	0.0019
X_8_	Quantile	5	0.1431	0.4815
X_9_	Natural Breaks (Jenks)	5	0.6237	0.0051
X_10_	Natural Breaks (Jenks)	5	0.7256	0.0001
X11	Quantile	5	0.2258	0.2816

The q-value indicates the ​​explanatory power of factors​​, while the p-value reflects the ​​statistical significance​​ of this explanatory power

### Interaction detection analysis of the influencing factors for elderly care institutions

**Fig. 7:**
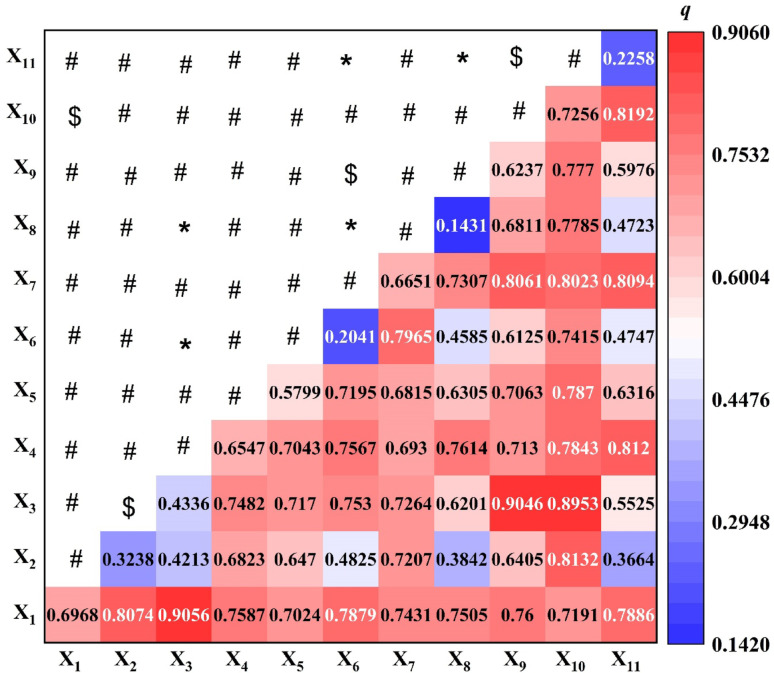
Interaction detection results of influencing factors for the elderly care institutions. (Note: **#** denotes two-factor enhancement, * denotes nonlinear weakening, **$** denotes single-factor nonlinear weakening [26, 27], and the letters X_1_, X_2_,., X_11_ represent factor names (for details, see Fig. 6))

Interaction detection analysis was conducted to evaluate whether the pairwise interactions between influencing factors amplify or diminish their effects on the spatial distribution of ECIs [26, 27]. The results, summarized in Fig. 7, indicate that interactions between the 11 variables mainly produced three types of effects: two-factor enhancement, nonlinear enhancement, and single-factor nonlinear weakening. Notably, the interactions between the following factor pairs exhibited enhancement, with explanatory power (*q*-value) exceeding 80%: resident population ∩ old-age dependency ratio, resident population ∩ proportion of population aged 65 and above, old-age dependency ratio ∩ general public budget expenditure, old-age dependency ratio ∩ number of hospital beds, public transportation condition ∩ number of hospital beds, number of hospital beds ∩ urbanization rate, public transportation condition ∩ general public budget expenditure, and public transportation condition ∩ urbanization rate. Furthermore, Fig. 7 indicates that the interaction between resident population or number of hospital beds and any other factor consistently resulted in an explanatory power above 70%, suggesting that these two factors are the most significant drivers of the spatial distribution of ECIs. Similarly, interactions involving public transportation condition and other factors all yielded explanatory power above 68%, underscoring its role as another major influencing factor. Meanwhile, instances of nonlinear weakening and single-factor nonlinear weakening were also observed. Specifically, the interactions between old-age dependency ratio ∩ GDP per capita and old-age dependency ratio ∩ disposable income per capita were characterized primarily by nonlinear weakening. In contrast, the interactive effects between resident population ∩ number of hospital beds, proportion of population aged 65 and above ∩ old-age dependency ratio, GDP per capita ∩ general public budget expenditure, and general public budget expenditure ∩ urbanization rate resulted in lower explanatory power (*q*-value) than that of the individual factors alone, indicating no enhancement in explaining the spatial distribution.

Combined with the factor detection results, it can be concluded that resident population and number of hospital beds are the dominant factors influencing the spatial distribution of ECIs. Their interactions with other factors significantly improve the explanatory power of the model regarding the spatial distribution, highlighting that the spatial pattern of these institutions is fundamentally shaped by interregional disparities in resident population. Nevertheless, the actual number of institutions in each province or city remains contingent upon local socioeconomic development conditions. In other words, the spatial distribution of ECIs arises from the dynamic coupling of multiple dimensions, including population size, medical resources, economic strength, and policy interventions. Resident population and medical resources form the core supporting framework of the spatial layout, while other factors amplify or suppress this layout through interactive effects. Looking forward, a coordinated mechanism of “precision-demand identification – graded policy compensation – regional resource sharing” should be adopted to address the polarizing dilemma wherein developed regions benefit from resource siphoning while less-developed areas suffer from inefficient service delivery. This approach will facilitate a shift in the resource allocation of ECIs from passively adapting to demand to actively optimizing suitability.

## Discussion

This study systematically reveals the spatial distribution patterns, equilibrium characteristics, and driving factors of ECIs across China through a multi-dimensional geographical perspective. The findings confirm significant spatial heterogeneity and imbalance in the provision of ECIs at the national scale, providing an empirical basis for optimizing the allocation of elderly care resources and promoting coordinated regional development.

### The core-periphery spatial pattern and its formation mechanism

This study identifies a distinct “core-periphery” hierarchy in China’s ECIs spatial distribution, closely aligned with the “Hu Huanyong Line” and exhibiting a “*T*”-shaped agglomeration pattern. While provinces such as Henan, Sichuan, and Anhui account for 34.1% of national ECIs, four western provinces/regions and Hainan contribute only 1.6% (Fig. 1). This pattern reflects underlying socioeconomic disparities and physical geography [10, 31]. Core zones like Shanghai and Chongqing benefit from economic agglomeration, aging populations, and policy support, whereas western regions face ecological fragility, sparse populations, and fiscal constraints. Notably, per capita ECI availability shows distinct regional characteristics: the three northeastern provinces maintain high supply levels (> 0.54 per 10,000 people) due to accelerated aging from youth out-migration and a large existing institutional base, while economically developed Guangdong has low supply (< 0.14) because of land scarcity and large youth immigrant populations. This suggests that ECI supply is not solely determined by economic strength but by the combined effect of aging pressure, resource constraints, and population structure. This polarized spatial pattern reflects the “Matthew effect” in the allocation of elderly care resources, where developed regions attract more resources, potentially leading to an oversupply of high-end services, while less developed areas remain trapped in “low-level equilibrium” with insufficient basic service provision [16, 31]. Additionally, significant urban-rural disparities merit attention, with urban ECIs demonstrating substantially higher development levels than their rural counterparts [32, 33]. This spatial disparity challenges China’s goal of ensuring equitable access to basic elderly care. In response, the Central Committee of the CPC and State Council have prioritized aging and livelihood issues through a dual-track approach encompassing both strategy and implementation. The 20th CPC National Congress outlined a national aging strategy emphasizing care service development and universal coverage, while the “14th Five-Year Plan for the National Aging Cause and Elderly Care System” designated public ECIs as safety-net providers for vulnerable groups [33].

To address resource mismatches, we propose spatially differentiated measures based on national guidelines [4]. In eastern hotspots like the Yangtze River Delta, policies should incentivize private capital into integrated medical-care models to resolve “supply lagging behind demand”; in western cold spots, targeted fiscal transfers and regional sharing mechanisms are needed to advance equity, consistent with healthy aging objectives [34] and existing evidence [31]. Grounded in empirical findings—such as the role of permanent population and hospital beds—these recommendations represent a transition from subjective proposals to evidence-informed, policy-integrated solutions.

### Interpretation of core findings

Three principal findings emerge from this study, together illustrating how demographic, economic, and policy factors jointly shape the geography of ECIs in China.

First, the spatial distribution of ECIs exhibits a “core-periphery” hierarchy that aligns with the “Hu Huanyong Line,” forming a " *T* “-shaped agglomeration pattern. Over one-third of national institutions are concentrated in six provinces including Henan and Sichuan, whereas four western provinces/regions and Hainan account for only 1.6% (Fig. 1). This reflects fundamental demographic and economic gradients, with eastern regions benefiting from stronger economic foundations while the other regions are constrained by fragile ecology, sparse populations, and limited fiscal capacity.

Second, the distribution shows significant spatial agglomeration and supply-demand mismatch. A “*T*”-shaped cluster of high-density cores—anchored by Shanghai and Chongqing—aligns with major economic corridors (Fig. 3). The consistency coefficient (*CC*) reveals a structural imbalance: border and northeastern regions show supply exceeding demand localized demand (CC > 1), benefiting from national policies, while eastern coastal and central provinces exhibit the opposite (CC < 1) due to aging pressures and land constraints (Fig. 4b). This polarization is corroborated by the imbalance index (*S* = 0.39) and hot-cold spot analysis, which together illustrate a “Matthew effect” in resource allocation (Fig. 5).

Third, permanent population size and hospital bed availability emerge as dominant factors influencing ECI distribution. Their interactions with other factors (e.g., public transportation, fiscal expenditure) significantly enhance the explanatory power (*q*-value > 70%; Fig. 7). This finding highlights medical resources as a “rigid constraint " in ECI planning, which is particularly relevant for serving China’s 35 million disabled and semi-disabled older adults [35]. In contrast, economic factors like GDP and per capita disposable income show weaker explanatory power (*q*-value < 30%), reflecting the current ECI model’s reliance on government support and universal services rather than market mechanisms alone.

### Limitations and future research directions

This study has several limitations that suggest productive pathways for future research. First, the provincial-level scale of analysis may obscure significant intra-provincial disparities. Future research should adopt finer spatial scales—such as municipal or county levels—and incorporate Point of Interest (POI) data to uncover such micro-scale distribution patterns [12, 15]. Second, the reliance on permanent population data limits our ability to capture how inter-regional population flows dynamically reshape elderly care demand. This static approach likely leads to a systematic underestimation of demand pressure in aging, net out-migration provinces, while obscuring the latent future demand in younger, net in-migration provinces. To address this, subsequent work will leverage high-resolution mobility data (e.g., mobile phone signaling, Baidu Migration data) to build an origin-destination demand model, thereby enabling more accurate forecasting and strategic allocation of regional elderly care resources. Finally, while this study concentrates on the quantity of ECIs and socioeconomic determinants, it does not incorporate important qualitative aspects—such as service quality—or other potential influences such as local policy intensity or cultural attitudes toward institutional care. Subsequent studies would benefit from integrating quality-related indicators and a wider range of factors to enable a more holistic understanding of disparities in elderly care resources.

## Conclusions

This study systematically examines the spatial distribution, supply-demand dynamics, and driving factors of ECIs in China, leading to the following core conclusions. ECIs show a “core-periphery” hierarchy and “*T*”-shaped agglomeration, aligning with the “Hu Huanyong Line.” Regional disparities are evident: over one-third cluster in six southeastern provinces, versus just 1.6% in other regions, shaped by demographic distribution, socioeconomic, and geographic disparities. The supply of ECIs and the aging population face dual challenges of spatial mismatch and structural imbalance. Northeastern provinces show “supply ahead of demand,” aided by policy support, whereas high-demand economic zones (e.g., the Yangtze River Delta) face supply shortfalls. The Imbalance Index (S = 0.39) and hot-cold spot analysis confirm a “Matthew effect”: developed regions draw more resources, while underdeveloped areas stay trapped in low-level equilibrium. The OPGD identifies permanent population size and hospital bed availability as dominant factors of ECI distribution. Their interactions with factors like fiscal expenditure significantly enhance the explanatory power, highlighting resources as a “rigid constraint” in ECI planning. GDP and income exert weaker influences, reflecting ECI development’s reliance on government support over market mechanisms.

In summary, the spatial pattern of ECIs in China result from dynamic coupling of population, medical resources, economy, and policy. Addressing imbalances requires coordinated precision demand assessment, differentiated policies, and resource sharing—shifting from passive adaptation to active optimization in elderly care resource allocation.

## Data Availability

No datasets were generated or analysed during the current study.
